# Identification and validation of the biomarkers related to ferroptosis in calcium oxalate nephrolithiasis

**DOI:** 10.18632/aging.205684

**Published:** 2024-03-25

**Authors:** Chao Hou, Bing Zhong, Shuo Gu, Yunyan Wang, Lu Ji

**Affiliations:** 1 Department of Urology, The Affiliated Huai'an First People’s Hospital of Nanjing Medical University, Huai’an 223300, Jiangsu, China

**Keywords:** ferroptosis, biomarkers, calcium oxalate nephrolithiasis, bioinformatics

## Abstract

Ferroptosis is a specific type of programmed cell death characterized by iron-dependent lipid peroxidation. Understanding the involvement of ferroptosis in calcium oxalate (CaOx) stone formation may reveal potential targets for this condition. The publicly available dataset GSE73680 was used to identify 61 differentially expressed ferroptosis-related genes (DEFERGs) between normal kidney tissues and Randall's plaques (RPs) from patients with nephrolithiasis through employing weighted gene co-expression network analysis (WGCNA). The findings were validated through *in vitro* and *in vivo* experiments using CaOx nephrolithiasis rat models induced by 1% ethylene glycol administration and HK-2 cell models treated with 1 mM oxalate. Through WGCNA and the machine learning algorithm, we identified LAMP2 and MDM4 as the hub DEFERGs. Subsequently, nephrolithiasis samples were classified into cluster 1 and cluster 2 based on the expression of the hub DEFERGs. Validation experiments demonstrated decreased expression of LAMP2 and MDM4 in CaOx nephrolithiasis animal models and cells. Treatment with ferrostatin-1 (Fer-1), a ferroptosis inhibitor, partially reversed oxidative stress and lipid peroxidation in CaOx nephrolithiasis models. Moreover, Fer-1 also reversed the expression changes of LAMP2 and MDM4 in CaOx nephrolithiasis models. Our findings suggest that ferroptosis may be involved in the formation of CaOx kidney stones through the regulation of LAMP2 and MDM4.

## INTRODUCTION

Calcium oxalate (CaOx) nephrolithiasis occurs with a high recurrence rate, posing financial hardship for individuals and society [1]. Despite the progress in surgical techniques to improve the efficiency of stone removal, the drugs for nephrolithiasis have been impeded due to the lack of full understanding of the pathogenesis [2]. It is widely accepted that supersaturation and the build-up of CaOx crystals in renal tissues are the main causes of apoptosis and damage to the kidney tubular cells, a process that can lead to the continual occurrence of kidney stones [3]. Randall’s plaques (RPs) concept is an accepted theory that is used to explain CaOx stone formation [4]. It is believed that RPs can serve as a nidus, or a starting point, for the formation of CaOx stones, in which plaques provide a rough surface where CaOx crystals can anchor and grow, eventually leading to stone formation [5]. By understanding the mechanisms underlying kidney stone formation, new diagnostic or therapeutic strategies may be developed for CaOx nephrolithiasis.

Ferroptosis is a type of cell death via iron-dependent lipid peroxides [6]. During ferroptosis, dysregulation of cellular iron metabolism leads to increased levels of labile iron and highly reactive hydroxyl radicals [7]. Lipid peroxidation (LPO) is a key driver of ferroptosis, leading to the production of lipid peroxides, further propagating the oxidative damage, cellular dysfunction, and eventual cell death [8, 9]. This process is regulated by several key players such as glutathione peroxidase 4 (GPX4), which functions to reduce lipid peroxides and prevent ferroptosis [10, 11]. Ferroptosis is involved in diverse kidney diseases, such as glomerular disease [12], acute kidney injury (AKI) [13], diabetic kidney disease [14] and so on. In CaOx nephrolithiasis, studies have demonstrated that accumulation of lipid peroxides can contribute to the initiation of ferroptosis in renal cells, further resulting in cell death pathways, which is the common trigger for CaOx stone formation [15]. Also, the LPO markers, such as malondialdehyde (MDA) and glutathione (GSH) were reported to increase in CaOx nephrolithiasis patients [16, 17]. The interplay of ferroptosis in CaOx nephrolithiasis is an area of ongoing research, but the comprehensive role of ferroptosis in CaOx nephrolithiasis has not been explored based on the transcriptome data of nephrolithiasis patients.

In this study, we initially utilized the GSE73680 dataset to compare gene expression profiles between normal renal papillary tissues and RPs from patients with nephrolithiasis. By employing weighted gene co-expression network analysis (WGCNA) and a machine learning algorithm, we identified the hub differentially expressed ferroptosis-related genes (DEFERGs), namely LAMP2 and MDM4. Based on the expression patterns of the hub DEFERGs, we divided the nephrolithiasis patients into two separate clusters and then conducted the functional enrichment analysis. Furthermore, we assessed the markers of ferroptosis within the two clusters. To validate our findings, we performed experiments with CaOx nephrolithiasis animal and cell models, specifically focusing on the expression of LAMP2 and MDM4. Our research will bring a new understanding of the impact of ferroptosis in nephrolithiasis and should offer potential biomarkers for CaOx nephrolithiasis.

## MATERIALS AND METHODS

### Data analysis

The GSE73680 dataset was employed in this study, consisting of 6 normal renal papillary tissues from individuals without a history of kidney stones (referred to as the “Normal” group) and 29 RPs from patients with calcium stones (referred to as the “Plaque” group). Then, 484 ferroptosis-related genes were achieved from the FerrDb database (http://www.zhounan.org/ferrdb/current/) (Supplementary Table 1). A Venn plot was generated to display the intersection between these ferroptosis-related genes that were differentially expressed in the GSE73680 dataset. Among these, we identified 61 genes as differentially expressed ferroptosis-related genes (DEFERGs) based on the criteria of |Log2 FoldChange|≥1 and *P* < 0.05 (Supplementary Table 2).

### Weighted gene co-expression network analysis

We first computed pairwise correlations between all genes in the dataset to establish the basis for constructing the co-expression network. Then, using the formula amn = |cmn|β, where cmn represents Pearson’s correlation between gene m and gene n, we constructed a weighted adjacency matrix. The parameter β determined the soft threshold power value. Next, we calculated different β values to assess the scale-free topology fit index and chose the one that yielded the best scale-free network. Following, the topological overlap measure (TOM) matrix quantifies the network connectivity of a gene by summing its adjacency values with all other genes in the network. Subsequently, genes with similar expression profiles were grouped into gene modules with average linkage hierarchical clustering. Afterwards, the gene significance (GS), and module membership (MM) were calculated for each gene with clinical traits. The GS represents the correlation between gene expression and the clinical trait, while the MM quantifies the degree of connectivity of a gene within its module. The nephrolithiasis-related modules were determined by assessing the coefficient of determination (R^2^) and statistical significance (P < 0.05). Finally, we identified 22 key DEFERGs within the nephrolithiasis-related modules.

### Machine learning

To screen the hub DEFERGs, a machine learning algorithm was employed. Specifically, the least absolute shrinkage and selection operator (LASSO) regression approach was utilized with “glmnet” R package. Through the LASSO analysis, two DEFERGs were identified as the hub DEFERGs based on their importance and contribution to the model. To assess the discriminatory capacity of the two hub DEFERGs in distinguishing between Plaque group and Normal group, a receiver operating characteristic (ROC) curve was built to discriminate between these groups.

### Consensus clustering and functional enrichment analysis

RPs samples were grouped under an unsupervised hierarchical clustering analysis with the “ConsensusClusterPlus” R package. To ascertain the optimal number of clusters, consensus matrix plots, cumulative distribution function (CDF) plots and the relative change in area under the CDF curve were employed. Additionally, principal component analysis was used to evaluate the disparities between clusters. The “limma” R package was utilized to identify differentially expressed genes (DEGs) between clusters, with a threshold of |log2(fold change)| > 3 and False discovery rate (FDR) < 0.01. Gene Ontology (GO) enrichment analysis and Kyoto Encyclopedia of Genes and Genomes (KEGG) pathway analysis were conducted with an adjusted *P* < 0.05. To further explore significant functional differences between clusters, we employed gene set enrichment analysis (GSEA) and Gene Set Variation Analysis (GSVA) with “clusterProfiler” R package by FDR < 0.25 and *P* < 0.05, allowing for the evaluation of enrichment of predefined gene sets or pathways within the clusters.

### CaOx stone rat model and treatment

Male Sprague-Dawley rats aged 5-6 weeks were divided equally with n = 6 into three groups: the control group, the CaOx group, and the ferrostatin-1 (Fer-1) group. The control group had access to water ad libitum, while the CaOx group received 1% ethylene glycol (EG) in their drinking water. The Fer-1 group was treated with 1% EG in their drinking water and received an additional gavage administration of 0.25 mg/kg/d of Fer-1. Fer-1 was initially dissolved in dimethyl sulfoxide (DMSO) and then further diluted with 0.9% NaCl solution. The resulting solution had a final concentration of 0.25 mg/ml for Fer-1 and 0.1% for DMSO, which was administered via gavage to the Fer-1 group. Meanwhile, the control group and the CaOx group were gavaged with equal volumes of 0.9% NaCl solution containing 0.1% DMSO. After four weeks, blood plasma and renal tissues were collected for further analysis.

### Histologic analysis

Various histologic staining techniques were employed to visualize specific components and proteins in the renal tissues. Hematoxylin and eosin (HE) staining was performed to observe the general structures of the renal tissues. Von Kossa (VK) staining was used to detect calcium deposits, while diaminobenzidine (DAB)-enhanced Prussian blue (PB) staining was utilized to identify iron deposits. Trivalent iron in tissues can form a blue precipitate with potassium ferricyanide, indicating a high content of iron elements in the tissue. Subsequent addition of DAB triggers an oxidation-reduction reaction between DAB and the precipitated trivalent iron, forming a brown compound, which improves the specificity of detection. Masson staining was performed to visualize collagen fibers and assess fibrosis in the renal tissues. Immunohistochemistry (IHC) and immunofluorescence (IF) were applied to detect the expression levels and localization of specific proteins in the renal tissues. Detailed information about the antibodies can be found in Supplementary Table 3. A blind histological evaluation of all sections stained was undertaken by two independent investigators to prevent bias. Either the person conducting the histologic analysis or the individual providing the samples is unaware of certain information.

### Cell culture, cell counting kit-8 (CCK-8) and lactate dehydrogenase (LDH) assay

The HK-2 cell line, sourced from the Chinese Academy of Sciences in Shanghai, China, was subjected to various treatments. All the initial HK-2 cells were seeded equally in each case after counting with trypan blue. Since the effect of 1 mM oxalate on HK-2 cells for 24h was the most significant, we chose 1 mM concentration to carry out the follow-up experiments [18, 19], while a subset of HK-2 cells, designated as the Fer-1 group, received a 2-hour pretreatment with Fer-1 (2 μM). After accurately calculating the number of cells with trypan blue, 3 × 10^3^ HK-2 cells from different groups were seeded in a 96-well plate. Subsequently, 10 μl of CCK-8 solution was added at the designated time and read at 450 nm absorbance. Cellular LDH activities were evaluated with the LDH Assay Kit (Cat# C0016, Beyotime, Shanghai, China) as per the manufacturer’s guidelines.

### Measurement of LPO and ROS levels

Malondialdehyde (MDA), glutathione (GSH), and superoxide dismutase (SOD) activities were quantified by MDA kits (Cat# A003-1), GSH kits (Cat# A005), and SOD kits (Cat# A001-1) (JianCheng, Nanjing, China), respectively. The levels of LPO of HK-2 cells in different groups were evaluated by Lipid Peroxidation Probe BDP 581/591 C11 (Cat# L267, Dojindo Laboratories, Japan) according to the manufacturer’s instructions. The assessment of reactive oxygen species (ROS) production was conducted using DCFH-DA and DHE staining, in accordance with the guidelines provided by the manufacturer, and was subsequently measured through microfluorimetry detection.

### Quantitative real-time PCR (qRT-PCR) and molecular docking

The total RNA from the renal tissues was extracted using the TRnaZol RNA Kit (Cat# M5102, NCM, Suzhou, China). qRT-PCR assays were performed using the SYBR Green PCR system (Cat# R311, Vazyme, Nanjing, China) independently replicated three times. The sequences of primers were also included in Supplementary Table 3. The structure of LAMP2 (PDB ID: 2MOF) and MDM4 (PDB ID: 3FDO) was obtained from the Protein Data Bank (PDB, https://www.rcsb.org/), while the structure of Fer-1 (PubChem CID: 4068248) was achieved from PubChem database (https://pubchem.ncbi.nlm.nih.gov/). Molecular docking was carried out using AutoDock Vina (version 1.1.2) for all calculations in this project as described in our previous study [18].

### Statistical analysis

The statistical analysis was conducted using R software (version 4.1.2) and GraphPad Prism (version 8.0). The Wilcox test, Student’s t-test, or ANOVA test were employed to compare two or more independent test series, respectively.

### Data availability statement

The original contributions presented in the study are included in the article/Supplementary Materials. Further inquiries can be directed to the corresponding author.

## RESULTS

### Identification of DEFERGs

**Figure 1:**
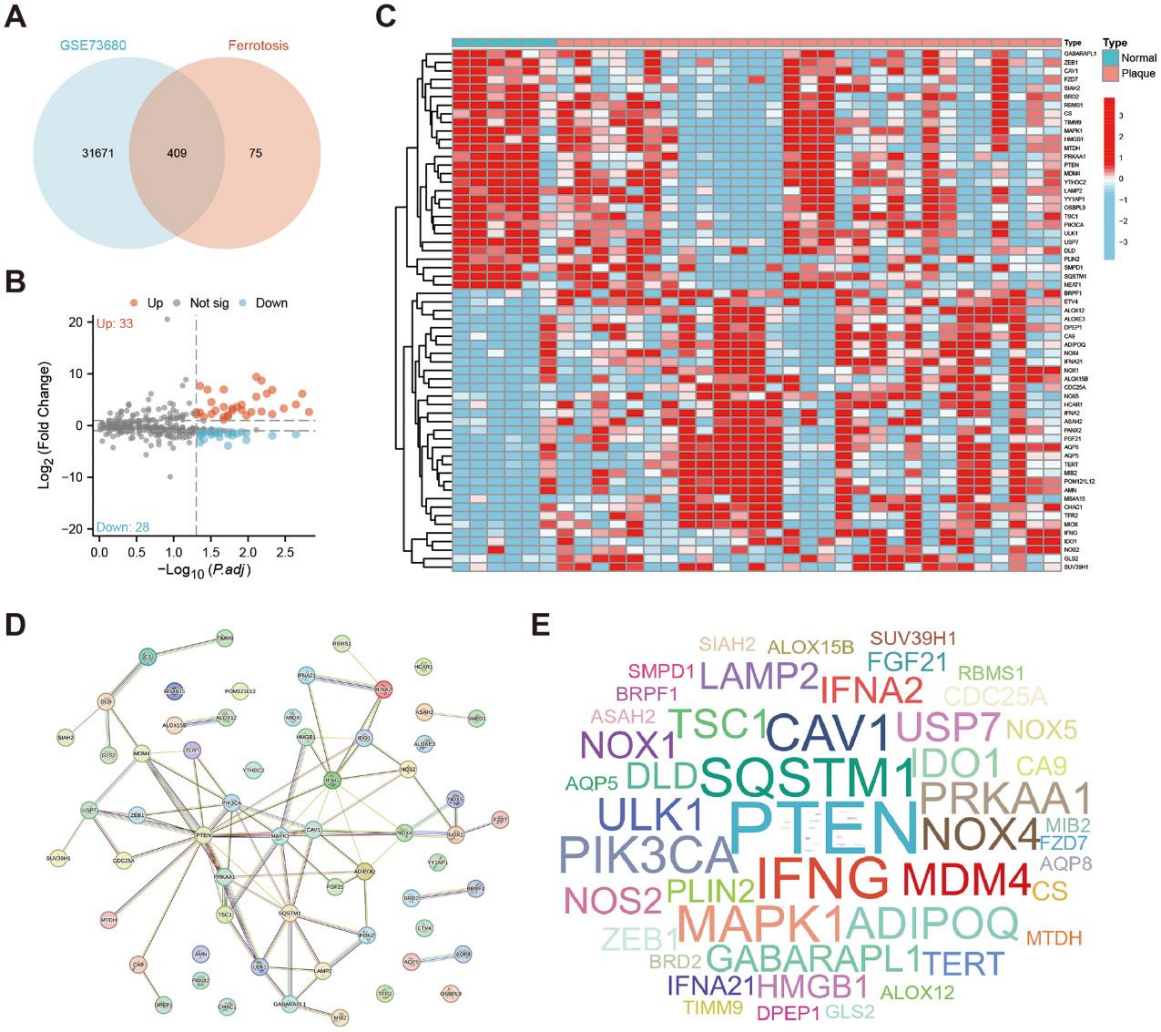
**Identification of DEFERGs.** (**A**) The Venn plot shows the number of ferroptosis-related genes that have intersections with the GSE73680 dataset (n=409). (**B**) The volcano plot of all DEFERGs between Normal and Plaque group (|Log_2_ FoldChange|≥1, adjusted *P* < 0.05). (**C**) The heatmap of DEFERGs between Normal and CaOx group. (**D**) PPI networks of the 61 DEFERGs. (**E**) The word-cloud map shows the scale of the adjacent nodes of DEFERGs, reflecting the importance of genes.

We first used the Venn plot to screen 409 ferroptosis-related genes that have intersections with the GSE73680 dataset (Figure 1A). With the threshold of |Log2 FoldChange|≥1 and adjusted *P* < 0.05, there are 61 ferroptosis-related genes identified as DEFERGs, and 33 of them were significantly upregulated in Plaque group, while 28 of them were significantly downregulated (Figure 1B). The heatmap showed the expression of these 61 DEFERGs in GSE73680 dataset (Figure 1C). An exploration into the protein-level connections between DEFERGs was conducted via the PPI network (Figure 1D). The word-cloud map showed the scale of the adjacent nodes of DEFERGs, reflecting the importance of PTEN, IFNG, SQSTM1, CAV1, MAPK1, PIK3CA, ADIPOQ, and MDM4 (Figure 1E). The adjacent nodes of DEFERGs were listed in Supplementary Table 4.

### Screening of the DEFERGs correlated with nephrolithiasis via WGCNA

**Figure 2:**
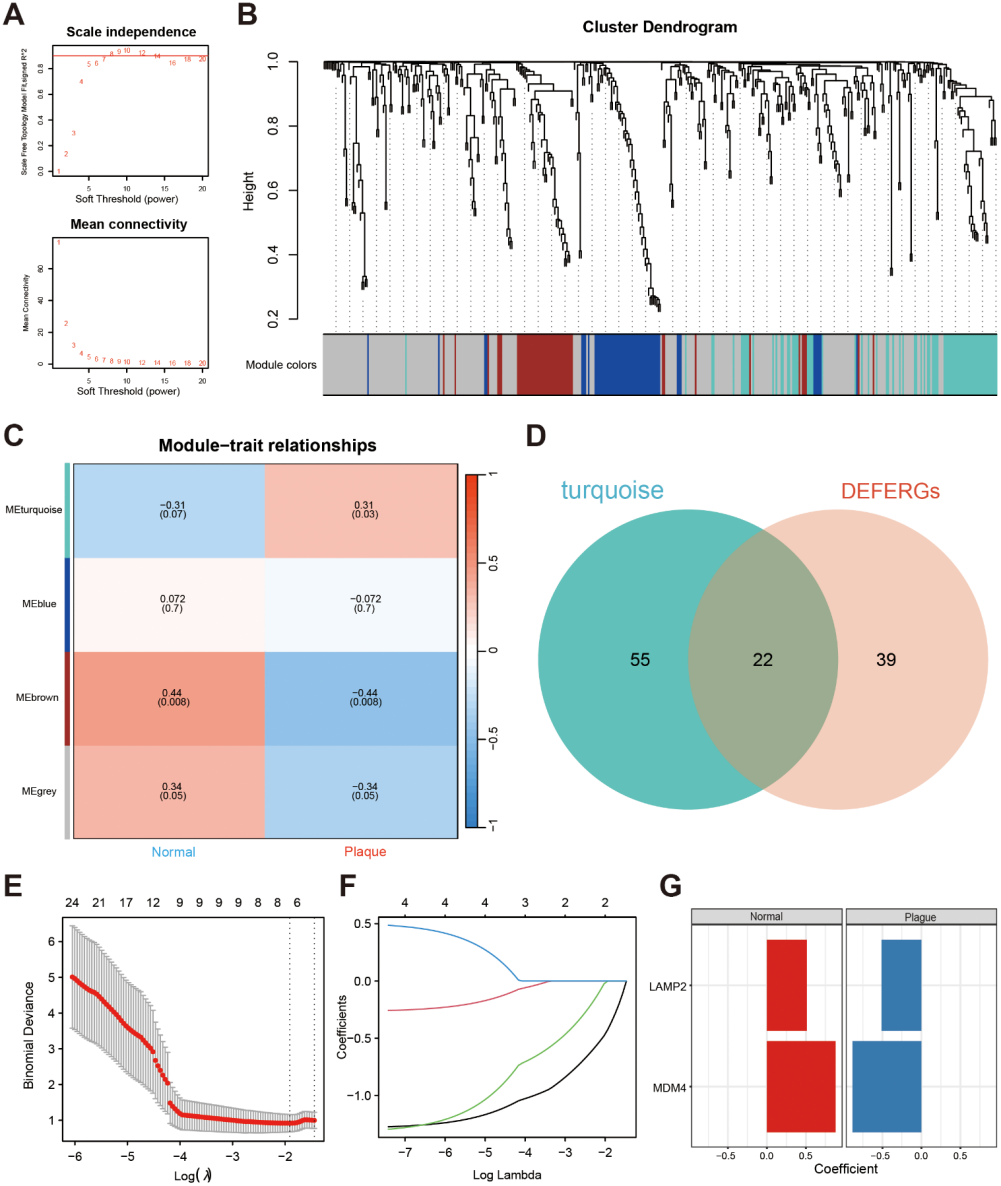
**Screening the hub DEFERGs via WGCNA and machine learning.** (**A**) The power value β, set at 8, is indicated by the red line, ensuring a robust R^2^ > 0.9. (**B**) The clustering dendrogram illustrates the amalgamation of gene co-expression modules, with each color signifying a distinct module. (**C**) The heatmap displays the correlation between modules and clinical traits, featuring correlation coefficients and corresponding P-values at the intersections of rows and columns. (**D**) The Venn plot illustrates the overlapping genes between DEFERGs and those found in the nephrolithiasis-related module (turquoise). (**E**, **F**) LASSO regression of the 22 intersected DEFERGs. (**G**) Coefficients between the two hub DEFERGs and the clinical portraits.

We employed WGCNA to identify modules associated with nephrolithiasis. We set the soft threshold power value β = 8 to construct a scale-free network (Figure 2A). Subsequently, a hierarchical clustering tree was generated and modules were detected by dynamic tree cutting. Four color modules were identified (Figure 2B), among which the turquoise module demonstrated a significantly positive correlation (r = 0.31, *P* = 0.03) with nephrolithiasis (Figure 2C). Gene expression matrix of the turquoise module via WGCNA analysis was listed in Supplementary Table 5. Further, 22 DEFERGs in the turquoise module were identified with the Venn plot, which might be related to nephrolithiasis (Figure 2D).

### Screening of the hub DEFERGs via machine learning

**Figure 3:**
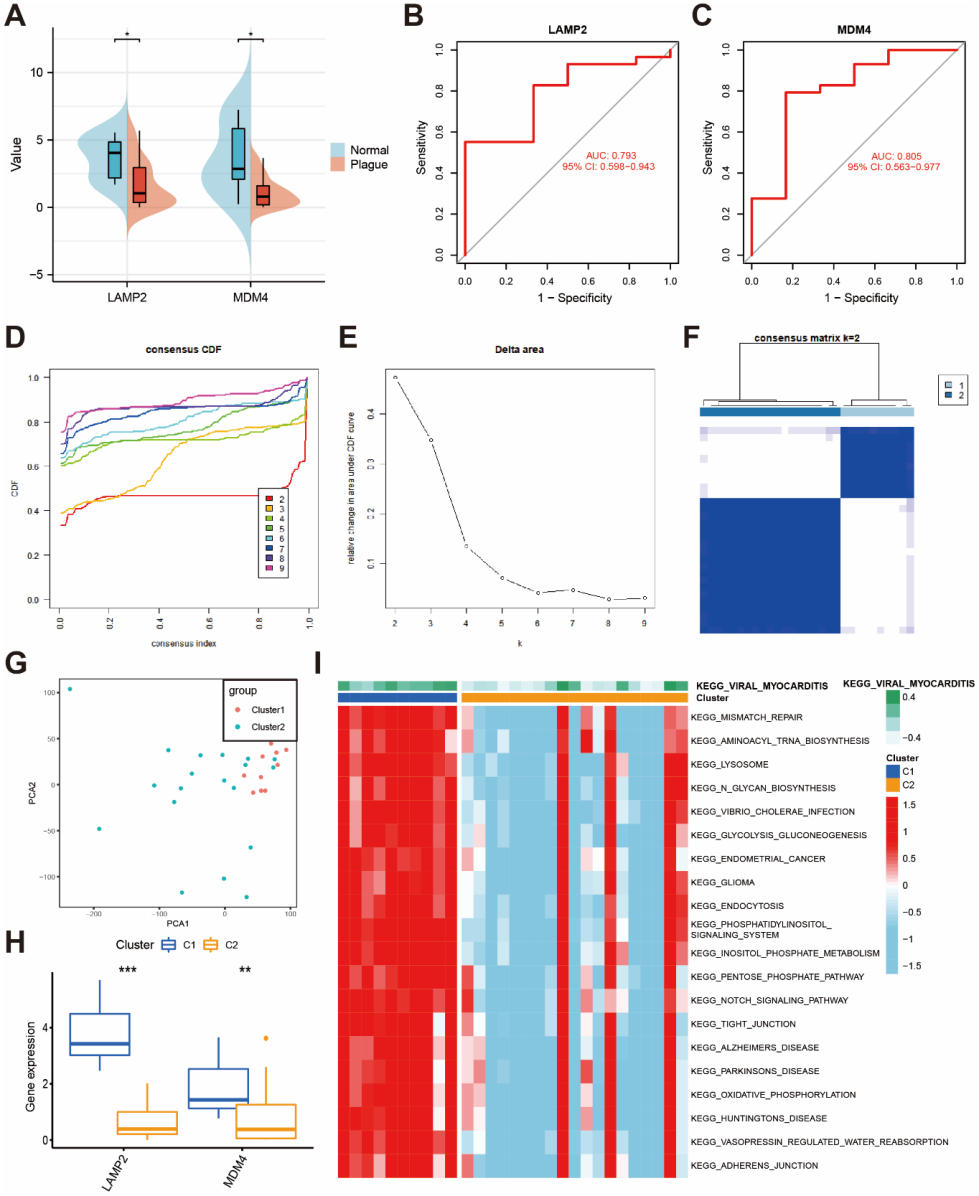
**Identification of two clusters based on hub DEFERGs.** (**A**) The bean plot shows the differential expression of hub DEFERGs between the normal tissues and RPs. (**B**, **C**) ROC curve of hub DEFERGs in RPs’ diagnosis. (**D**, **E**) The consensus cumulative distribution function (CDF) plot (**D**) and the relative change in area under the CDF curve (**E**). (**F**) The consensus matrix plot is displayed for k = 2 clusters. (**G**) The PCA plot visually represents the distribution of these two clusters. (**H**) Boxplots display the expression patterns of hub DEFERGs in cluster 1 and cluster 2. (**I**) GSVA analysis of biological pathways is conducted between the two distinct clusters, with red indicating activated pathways and blue indicating inhibited pathways.

We further employed LASSO regression analysis to identify potential hub genes associated with CaOx nephrolithiasis using the expression profiles of 22 DEFERGs (Figure 2E, 2F). LASSO regression analysis revealed two variables, LAMP2 and MDM4, as diagnostic markers for CaOx nephrolithiasis (Figure 2G). Subsequently, we observed a significant decrease in the expressions of LAMP2 and MDM4 in Plaque group (Figure 3A). To assess the diagnostic performance of LAMP2 and MDM4, we constructed ROC curves and obtained area under the curves (AUC) values of 0.793 and 0.805, respectively (Figure 3B, 3C), indicating that LAMP2 and MDM4 gene signatures have potential diagnostic values.

### Identification of two clusters based on the hub DEFERGs

The consensus clustering analysis of the expression profiles of LAMP2 and MDM4 revealed two distinct subtypes of CaOx nephrolithiasis. The stability of the clustering results was confirmed by the CDF curve (Figure 3D) and the CDF Delta area curve (Figure 3E). The consensus matrix plot showed that Plaque group could be divided into two clusters, namely cluster 1 (n = 10) and cluster 2 (n = 19) (Figure 3F). The detailed clusters based on the hub DEFERGs were shown in Supplementary Table 6. The PCA plot further demonstrated a clear distinction between the two clusters (Figure 3G). Additionally, the boxplot indicated that cluster 1 had higher expression of LAMP2 and MDM4 than cluster 2 (Figure 3H). As shown in Figure 3I, GSVA results revealed that cluster 1 was enriched in lipid metabolism, amino acid metabolism, bile acid metabolism, kidney metabolism, signal transduction, cell junctions, and immune response, which exert a major influence on CaOx nephrolithiasis.

### Functional distinctions between two clusters

**Figure 4:**
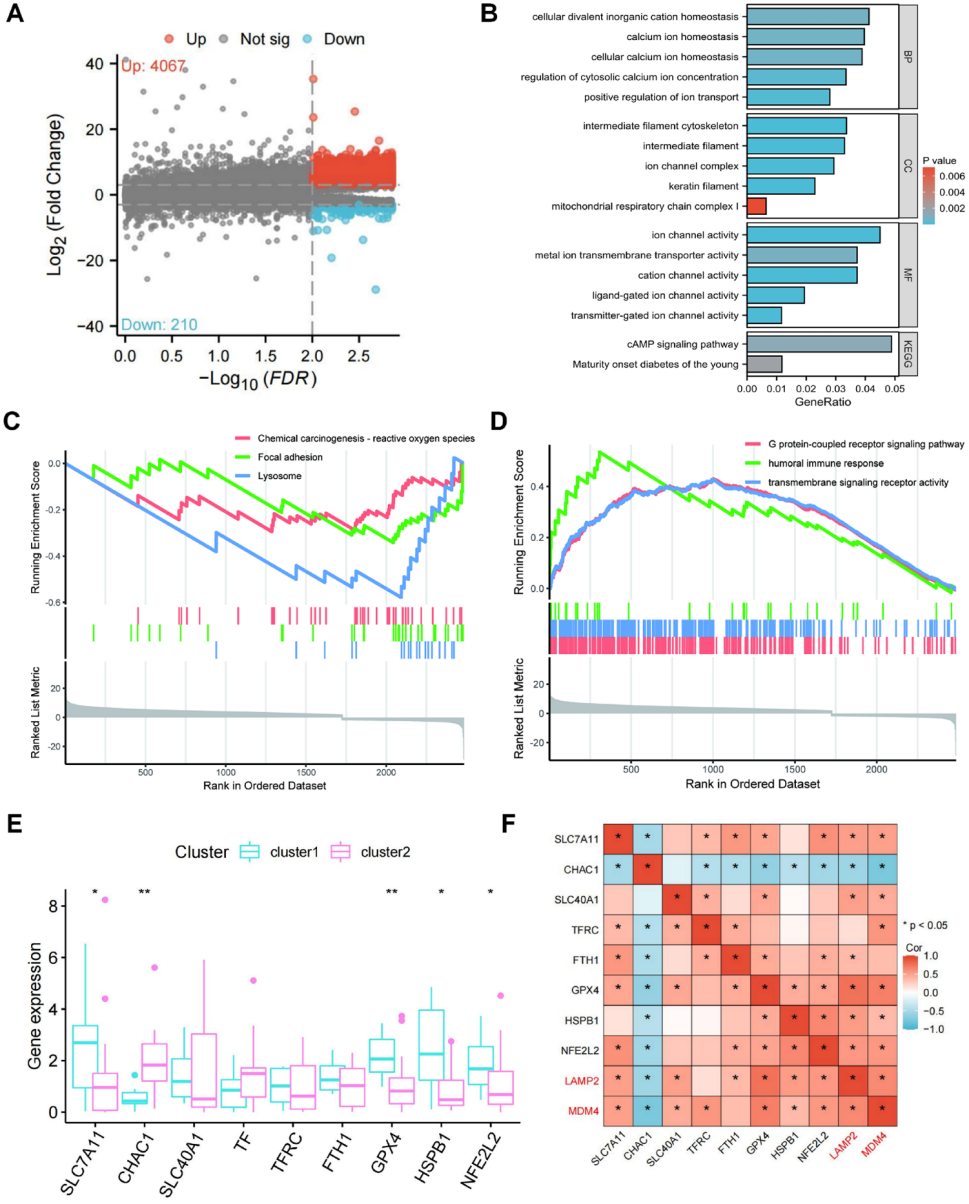
**Differentially expressed genes (DEGs) screening and pathway enrichment analysis between the two clusters.** (**A**) The volcano plot of all DEGs between two clusters. (**B**, **C**) GSEA analysis reveals GO terms (**B**) and KEGG terms (**C**) enriched by DEGs between the two clusters. (**D**) Enriched GO terms and KEGG pathways of DEGs. (**E**) Expression of the markers of ferroptosis in the two clusters. (**F**) Correlation of LAMP2 and MDM4 with the markers of ferroptosis.

A total of 4277 differentially expressed genes (DEGs) were identified between clusters (Supplementary Table 7). Compared to cluster 1, 4067 DEGs were upregulated and 210 DEGs were downregulated in cluster 2 (Figure 4A). G protein-coupled receptor signaling pathway, transmembrane signaling receptor activity, and humoral immune response were enriched in GO terms (Figure 4B). Lysosome, Focal adhesion, and Chemical carcinogenesis-reactive oxygen species were enriched in KEGG pathways (Figure 4C). As shown in Figure 4D, the GO analysis results, positive regulation of ion transport, regulation of cytosolic calcium ion concentration, cellular calcium ion homeostasis, calcium ion homeostasis, and cellular divalent inorganic cation homeostasis were significantly enriched in biological processes (BP); keratin filament, intermediate filament, intermediate filament cytoskeleton, ion channel complex, and mitochondrial respiratory chain complex I were significant involved in cellular components (CC); ion channel activity, cation channel activity, transmitter-gated ion channel activity, ligand-gated ion channel activity, and metal ion transmembrane transporter activity were significant included in molecular functions (MF). KEGG analysis demonstrated that the DEGs were enriched in cAMP signaling pathway, and Maturity onset diabetes of the young. These results suggest that the DEGs mainly participate in ion transport such as iron and calcium, which is closely related to ferroptosis and CaOx nephrolithiasis.

### Differences of ferroptosis markers between clusters

We further investigated the relationship between ferroptosis markers in two clusters, including SLC7A11, CHAC1, SLC40A1, TF, TFRC, FTH1, GPX4, HSPB1, and NFE2L2 (Figure 4E). The expression of ferroptosis suppressors (SLC7A11, GPX4, HSPB1, and NFE2L2) was higher in cluster 1 compared to cluster 2, while the expression of ferroptosis driver (CHAC1) was lower in cluster 1. Consistent with these, the heatmap revealed the positive correlation of LAMP2 and MDM4 with the ferroptosis suppressors, as well as the negative correlation of LAMP2 and MDM4 with the ferroptosis driver (Figure 4F). These results indicate that LAMP2 and MDM4 may inhibit ferroptosis in CaOx nephrolithiasis.

### Validation of the hub DEFERGs in CaOx nephrolithiasis rat models

**Figure 5:**
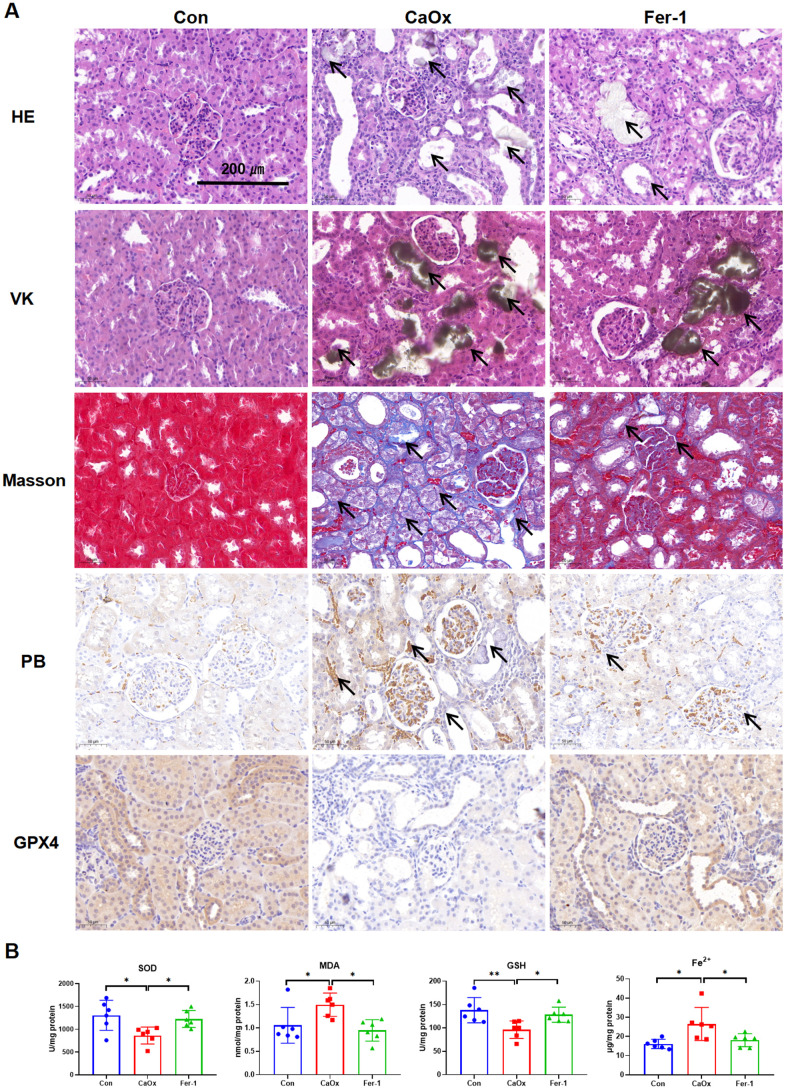
**Fer-1 reduces CaOx crystal deposition and kidney injury in CaOx nephrolithiasis rat models.** (**A**) Pathological sections including HE, VK, and Masson staining show the degree of kidney injury, crystal deposition, and kidney fibrosis; PB staining shows the Fe^3+^, while IHC staining shows GPX4 protein expression in different groups (magnification×20; scale bar, 200 μm). (**B**) SOD, MDA, GSH, and Fe^2+^ levels are detected in different groups (mean ± SD, n = 6, *P < 0.05, **P < 0.01).

To investigate the expression of LAMP2 and MDM4 in CaOx nephrolithiasis, the EG-induced CaOx nephrolithiasis rat models were constructed. The renal function results of rats showed that serum levels of blood urea nitrogen (BUN), serum creatinine (Scr), uric acid (UA), and Fe^3+^ in the CaOx model group were significantly elevated (Table 1). HE, VK, and Masson staining demonstrated the presence of tubular dilation and injury, CaOx crystals, and fibrosis in the renal tubules of the CaOx rats, which was reversed by the administration of Fer-1 (Figure 5A). PB staining showed significant iron accumulation in the CaOx group, and IHC results displayed a decrease in GPX4 expression in the CaOx group (Figure 5A). As depicted in Figure 5B, the Fer-1 group and the control group exhibited notably elevated levels of SOD and GSH in contrast to the CaOx group, whereas the MDA and Fe^2+^ levels displayed an inverse trend.

**Table 1:** Biochemical analysis of the levels of SCr, BUN, UA, Ca^2+^, and Fe^3+^ contents in serum of rats.

**Characteristics**	**Con (n=6)**	**CaOx (n=6)**	**Fer-1 (n=6)**
BUN (mg/dL)	5.01±0.61^**^	25.28±15.93	6.38±1.16^*^
Scr (μmol/L)	31.84±4.16^**^	71.88±29.48	44.58±4.21^*^
UA (μmol/L)	95.65±43.49^*^	192.82±79.99	102.97±39.21^*^
Ca^2+^ (mmol/L)	0.97±0.04^ns^	0.95±0.02	0.94±0.01 ^ns^
Fe^3+^ (μmol/L)	6.08±2.46^*^	16.53±8.62	8.06±3.37^*^

BUN, Blood urea nitrogen; Scr, Serum creatinine; UA, Uric acid; *P<0.05, **P<0.05, ns, not significant, compared with CaOx group.

**Figure 6:**
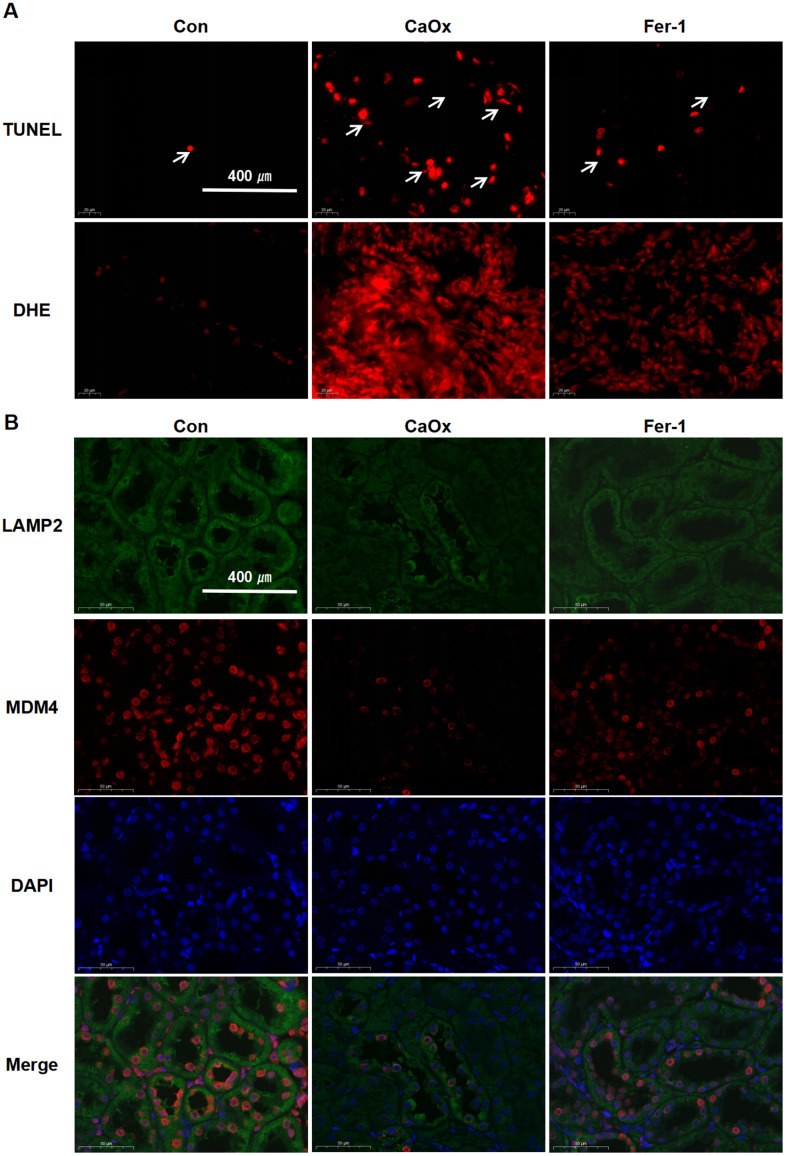
**Fer-1 reduces CaOx-induced LPO and apoptosis, as well as LAMP2 and MDM4 expression in CaOx nephrolithiasis rat models.** (**A**) TUNEL and DHE staining techniques are employed to evaluate apoptotic damage and the production of reactive oxygen species (ROS) in renal tissues (magnification×40; scale bar, 400 μm). (**B**) Representative immunofluorescence images show LAMP2 (green) and MDM4 (red) detection in renal tissues (magnification×40; scale bar, 400 μm).

TUNEL assay revealed that the apoptotic nuclear changes were markedly greater in the CaOx group than in the control and the Fer-1 group (Figure 6A). DHE fluorescence staining displayed the highest concentrations of intracellular oxidant species in the CaOx group (Figure 6A). Double-labelling IF results demonstrated that the protein expression of LAMP2 and MDM4 was attenuated in the CaOx group, which was in agreement with the bioinformatic analysis (Figure 6B). These findings demonstrate that the CaOx nephrolithiasis rat models were established successfully and Fer-1 treatment significantly reduced kidney injury, iron accumulation, and LPO in CaOx nephrolithiasis, suggesting the involvement of ferroptosis, as well as LAMP2 and MDM4 in CaOx nephrolithiasis.

### Validation of the hub DEFERGs in oxalate-induced HK-2 cell models

**Figure 7:**
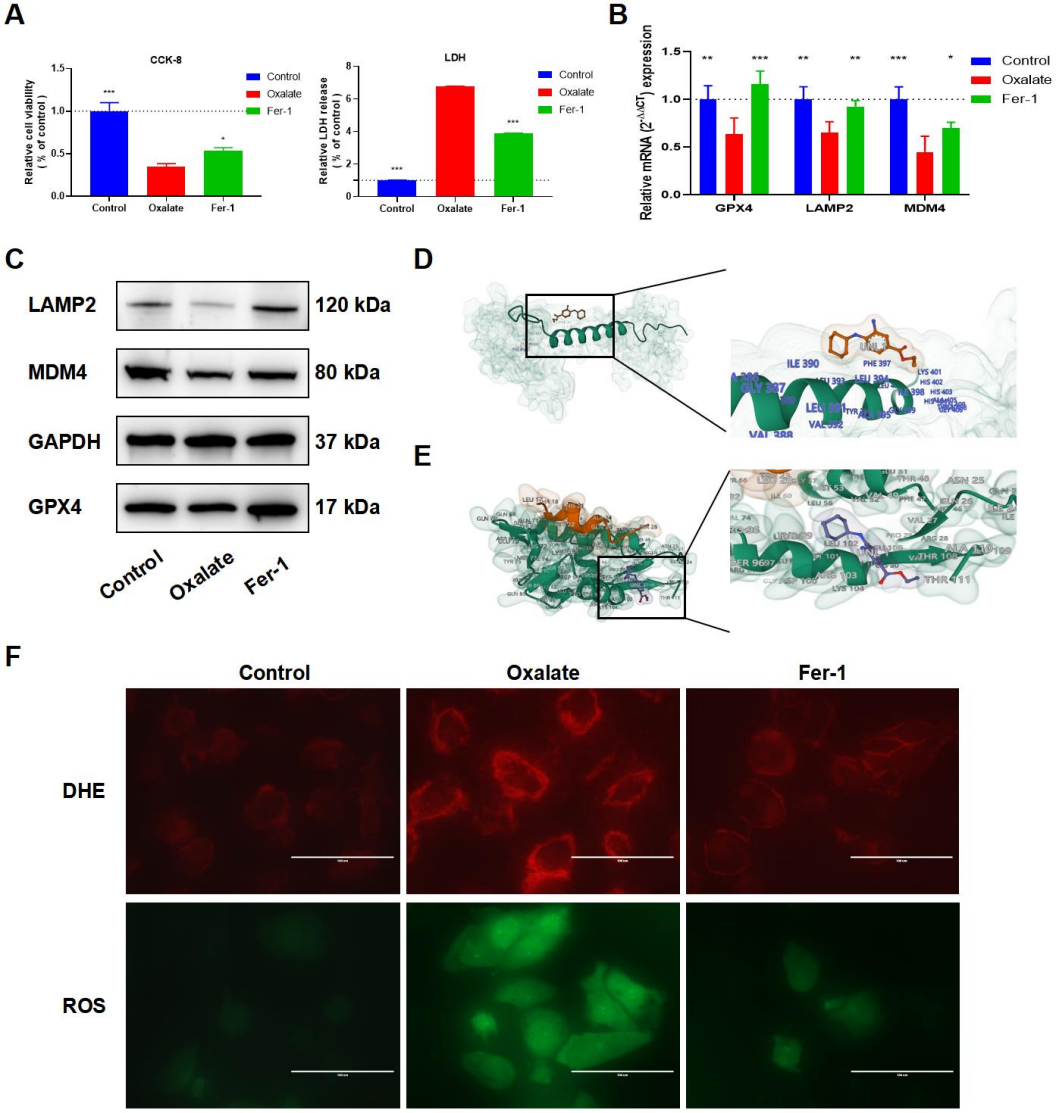
**Fer-1 suppresses oxalate-induced cytotoxicity and LPO, as well as LAMP2 and MDM4 expression in oxalate-induced HK-2 cells.** (**A**) Cell viability and LDH release after different treatment (mean ± SD, n = 3, *P < 0.05, ***P < 0.001). (**B**) qRT-PCR assays show GPX4, LAMP2, and MDM4 expression in HK-2 cells with different treatment (mean ± SD, n = 3, *P < 0.05, **P < 0.01, ***P < 0.001, compared with Oxalate group). (**C**) The LAMP2 and MDM4 protein expression of HK-2 cells in different groups. (**D**, **E**) Schematic diagram of Fer-1 docking with LAMP2 (**D**) and MDM4 (**E**). (**F**) Reactive oxygen species (ROS) generation in HK-2 cells was assessed using DHE and ROS staining (magnification×40; scale bar, 100 μm).

The cytoprotective effect of Fer-1 against cell injury induced by oxalate was evaluated using CCK-8 and LDH assays (Figure 7A). Furthermore, qRT-PCR (Figure 7B) and western blot (Figure 7C) analysis demonstrated that the mRNA and protein expressions of LAMP2 and MDM4 were decreased in the oxalate-treated group compared to the control group but were reversed after treatment with Fer-1. The binding energy results of molecular docking of Fer-1 with LAMP2 and MDM4 were -82.44 kcal/mol (LAMP2, Figure 7D), and -5.526 kcal/mol (MDM4, Figure 7E). Next, the DCFH-DA and DHE fluorescence intensity was observed to increase in the oxalate-treated group, but decreased following Fer-1 treatment (Figure 7F).

**Figure 8:**
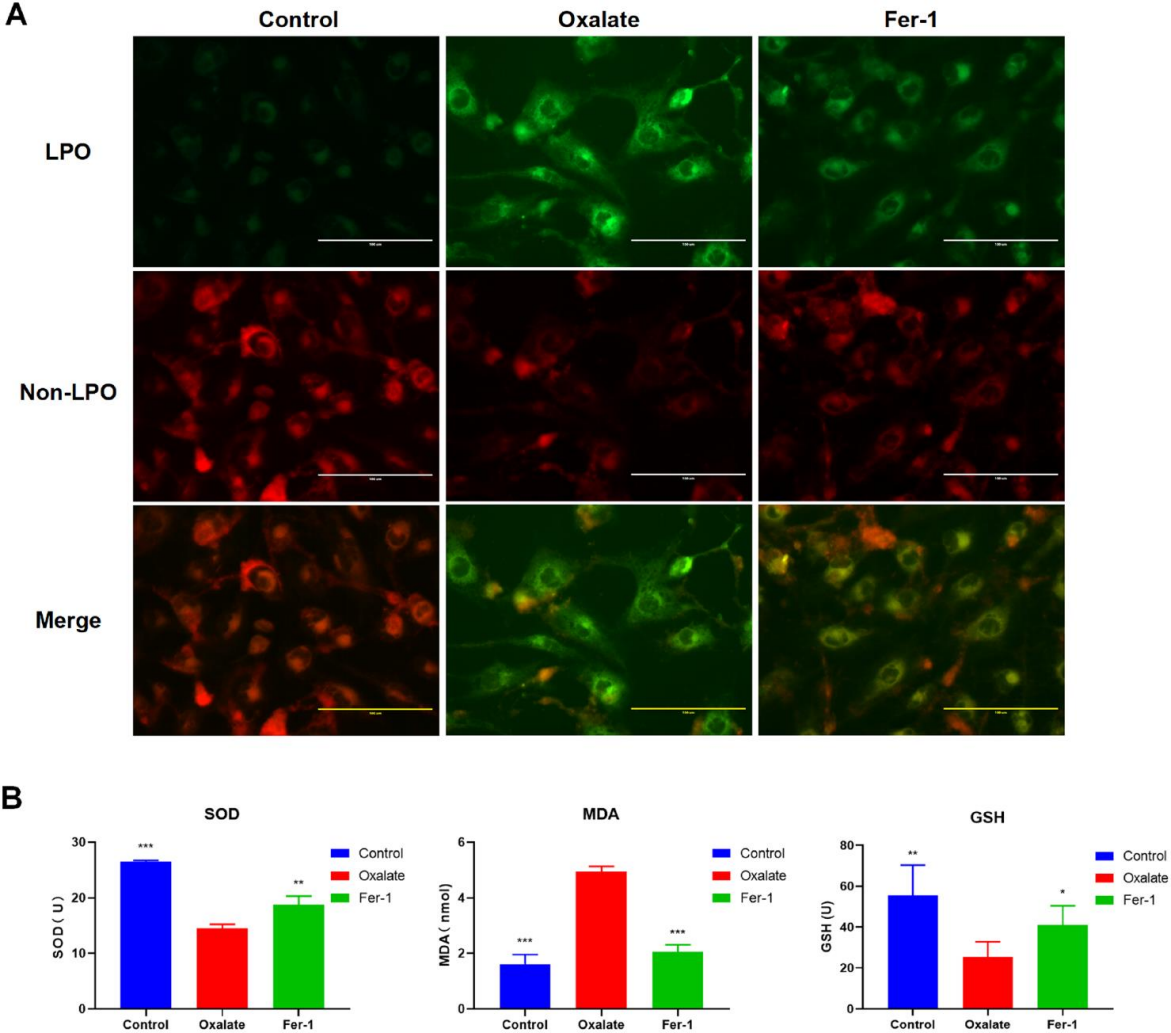
**Fer-1 suppresses oxalate-induced cytotoxicity and LPO in HK-2 cells.** (**A**) Measuring cellular LPO with the C11 BODIPY 581/591 fluorescent probe in HK-2 cells with different treatment (magnification×40; scale bar, 100 μm). (**B**) SOD, MDA, and GSH activity are detected in HK-2 cells with different treatment (mean ± SD, n = 3, *P < 0.05, **P < 0.01, ***P < 0.001, compared with Oxalate group).

Consistently, the results of LPO probe revealed that HK-2 cells treated with oxalate exhibited significantly elevated LPO level, but was also reduced after Fer-1 treatment (Figure 8A). Similarly, the MDA level was elevated in the oxalate-treated group, but the SOD and GSH levels were reduced. These findings indicate the potential of Fer-1 to protect human renal epithelial cells against oxalate-induced ferroptosis injury (Figure 8B). These data were consistent with bioinformatics screening, strongly suggesting that LAMP2 and MDM4 were potential biomarkers in CaOx nephrolithiasis.

## DISCUSSION

As a unique form of cell death, ferroptosis is a tightly regulated process involving the dysregulation of cellular iron homeostasis, accumulation of ROS, and LPO [20]. Meanwhile, ferroptosis is capable of altering inflammation and apoptosis, both of which are known to be closely associated with CaOx nephrolithiasis [4]. Understanding the molecular mechanisms underlying ferroptosis in CaOx nephrolithiasis may lead to the development of novel therapeutic strategies. Therefore, we systematically explored the role of ferroptosis in nephrolithiasis through the public dataset of nephrolithiasis patients (GSE73680).

Our initial analysis revealed 61 DEFERGs that differed between Normal and Plaque groups. Furthermore, we applied WGCNA and LASSO techniques to screen the hub DEFERGs, including LAMP2 and MDM4, which were decreased in Plaque group. The AUCs for both LAMP2 and MDM4 showed that they are reliable biomarkers for distinguishing Plaque group from Normal group. Meanwhile, our analysis found that LAMP2 and MDM4 were positively correlated with the ferroptosis suppressors (SLC7A11, GPX4, HSPB1, and NFE2L2), while were negatively correlated with the ferroptosis driver (CHAC1). As a key marker of ferroptosis, GPX4 prevents ferroptosis and catalyzes the reduction of lipid peroxides via converting harmful lipid peroxides into less harmful lipid alcohols [21]. Consistently, our qRT-PCR and IHC results displayed that GPX4 expression was significant down-regulation in CaOx nephrolithiasis models, while was relieved by Fer-1 treatment, suggesting the alteration of ferroptosis in CaOx nephrolithiasis.

The hub DEFERG, LAMP2, is a protein primarily located in the lysosomal membrane, playing a crucial role in lysosomal function and autophagy [22]. Alterations in LAMP2 expression and function have been observed in kidney diseases [23]. For example, Kain et al. earlier described LAMP2 as a target in patients with necrotizing glomerulonephritis [24]. Besides, studies have indicated that LAMP2 deficiency induced ferroptosis in retinal pigment epithelial cells [25]. Our bioinformatics analysis and experimental results both illustrated decreased expression of LAMP2 in CaOx nephrolithiasis. Consistent with our findings, Chaiyarit et al. discovered that LAMP2 was responsible for the degradation of CaOx crystals in renal tubular cells mediated by endolysosomes [26]. Another hub DEFERG, MDM4 (also known as MDMX) functions in cell growth and division via regulating the activity of p53 [27]. MDM4 interacts with p53 and inhibits its activity by preventing its binding to DNA and promoting its degradation [28]. MDM4 has been primarily studied in the context of ferroptosis. Venkatesh’s findings showed that inhibiting MDM4 could be beneficial in preventing degenerative diseases involving ferroptosis [29]. Similarly, inhibiting MDM2-MDM4 could induce ferroptosis and inhibit metastasis in pancreatic cancer [30]. Notably, the alterations in MDM4 expression, have been implicated in certain kidney disorders, such as kidney cancer [31, 32], AKI [33, 34], and else. Although limited research on the direct involvement of MDM4 in kidney stones, aberrations in the p53 pathway, have been implicated in CaOx stones [35]. Previous studies have discovered that p53 acted as a pro-apoptotic protein in CaOx stone with enhanced protein concentration in the stone animal group compared with the control group [36, 37]. However, LAMP2 and MDM4 are also very important in many other cell processes, and so their down-regulation is hardly a single mark of ferroptosis. Therefore, further research remains needed.

Moreover, we partitioned a set of 29 nephrolithiasis samples into two distinct clusters based on the expression levels of LAMP2 and MDM4. Analysis of GO and KEGG indicated that the DEGs between the two clusters primarily contribute to ion transportation processes such as iron and calcium, which are key regulators in ferroptosis and CaOx nephrolithiasis. Iron plays a crucial role in ferroptosis [38]. When iron levels become dysregulated or there is an imbalance in the cellular antioxidant defense system, it can lead to the accumulation of ROS, subsequent LPO, and the damage to cell membranes, which are key features of ferroptosis [39]. Meanwhile, calcium transport and CaOx nephrolithiasis are interconnected through the regulation of calcium and oxalate metabolism in the kidneys [40]. Further, we also performed GSEA analysis and the results suggested the DEGs might be associated with cell adhesion. Studies have revealed that cell junctions in renal tubular epithelial cells are essential in keeping the proper balance of ions and molecules in the urine, thus avoiding the formation of kidney stones [41]. Meanwhile, when the process of stone formation begins, CaOx can cause a decline in the intercellular cell junctions and the cells’ attachment to the basement membrane, and then cell junction barriers and fences are impaired, leading to renal tubulointerstitial injury [42]. In addition, GSVA results revealed that cluster 1 with higher expression of LAMP2 and MDM4, was enriched in lipid metabolism, amino acid metabolism, bile acid metabolism, and else, which can affect the risk of nephrolithiasis [43]. For example, abnormalities in lipid metabolism, specifically the metabolism of fats, subsequent obesity or insulin resistance, may contribute to the development and progression of kidney stones [44]. Regarding amino acid metabolism, oxalate is involved in glycine and hydroxyproline metabolism, and then elevated levels of oxalate in the urine potentially leads to CaOx stone formation [45]. These pathways analysis indicates the protective role of LAMP2 and MDM4 in CaOx nephrolithiasis, but further experimental validation is required.

Attention is being increasingly directed towards the part ferroptosis plays in nephrolithiasis. Recent studies have indicated that ferroptosis exerts a major performance in CaOx crystals [46]. It has been reported that Fer-1 could alleviate oxalate-induced renal tubular epithelial cell injury and CaOx stone formation by suppressing ferroptosis [47]. Similarly, Zhao’s study demonstrated that ANKRD1 promoted the formation of CaOx kidney stones by activating ferroptosis [48]. Moreover, inhibiting ferroptosis by Schizandrin B was reported to alleviate nephrolithiasis via Nrf2 signaling [49]. However, the comprehensive relationship between ferroptosis and CaOx nephrolithiasis has not been investigated, as well as the role of LAMP2 and MDM4. In this study, the function of ferroptosis was explored via public data analysis, and was confirmed by Fer-1 treatment in CaOx nephrolithiasis models. Fer-1 has been widely researched in connection to ferroptosis, and is found to be a highly effective inhibitor of this process by taking up lipid peroxyl radicals and cutting down on LPO [50, 51]. Next, the ferroptosis-related genes LAMP2 and MDM4 were verified in animals and cells via qRT-PCR, IF, or IHC assays, providing potential targets for the diagnosis and therapy of CaOx nephrolithiasis. Remarkably, the EG-induced rat model is commonly accepted and routinely employed in fundamental research on CaOx nephrolithiasis. However, criticism arises due to EG’s chronic toxicity [52]. Subsequent research, though, has indicated that EG administration at appropriate concentrations causes CaOx accumulation on tubular cell surfaces without harming other metabolic intermediates [53]. Consequently, most researchers continue to utilize EG as a standard lithogenic agent in rodent models [54]. Thus, we adopted this model in our study, ensuring its representativeness and reproducibility. Furthermore, we validated the impact on LAMP2 and MDM4, as well as the beneficial effects of Fer-1 in HK-2 cells, which are not linked to EG toxicity.

There are several limitations to consider in this study. Firstly, the precise role and underlying mechanisms of LAMP2 and MDM4 in CaOx nephrolithiasis remain unclear. Further validation and clarification through *in vitro* and *in vivo* experiments are necessary to establish specifical mechanisms. Secondly, the sample size of the public dataset used in the study was relatively small, emphasizing the need for larger sample sizes to ensure the robustness and generalizability of the findings. Thirdly, we do not study deeply whether overexpression of LAMP2 and MDM4 genes in HK-2 cells would decrease the cellular ferroptosis response to oxalate. Since it may be a therapeutic target for ferroptosis in calcium oxalate kidney stones, we plan to conduct some experiments to overexpress LAMP2 and MDM4 genes in HK-2 cells to determine the effects of these genes on the cellular response to ferroptosis.

In summary, we utilized WGCNA and a machine learning algorithm to identify two central ferroptosis-related genes (LAMP2 and MDM4) associated with CaOx nephrolithiasis. We further validated the involvement of ferroptosis and the expression of LAMP2 and MDM4 in animal models and cell lines of CaOx nephrolithiasis. Our findings provide a novel perspective on ferroptosis, as well as LAMP2 and MDM4, in the development of CaOx nephrolithiasis, offering potential targets for diagnosis and treatment strategies.

## Supplementary Material

Supplementary Table 1

Supplementary Table 2

Supplementary Tables 3 and 4

Supplementary Table 5

Supplementary Table 6

Supplementary Table 7
