# Supplementary material for: Identification and validation of the biomarkers related to ferroptosis in calcium oxalate nephrolithiasis
Source: Aging (Albany NY). 2024 Mar 25;16(7):5987–6007. doi: 10.18632/aging.205684 (PMC11042938; doi:10.18632/aging.205684)
Supplement: Supplementary Tables 3 and 4 [file aging-16-205684-s003.pdf]

SUPPLEMENTARY TABLES

Supplementary Table 3. The antibodies and primers used in this article.

Reagents	Vendor	Catalogue number	Information
Antibody-GPX4	ProteinTech	67763-1-Ig	IHC: 1:2000
Antibody-LAMP2	ProteinTech	66301-1-Ig	IHC: 1:1000, IF: 1:250
Antibody-MDM4	ProteinTech	17914-1-AP	IHC: 1:500, IF: 1:150
Primer-GPX4	Tsingke Biotechnology	/	Forward: 5'-GAGGCAAGACCGAAGTAAACTAC-3' Reverse: 5'-CCGAACTGGTTACACGGGAA-3
Primer-LAMP2	Tsingke Biotechnology	/	Forward: 5'-GCACAGTGAGCACAAATGAGT -3' Reverse: 5'-CAGTGGTGTGTATGGTGGGT -3
Primer-MDM4	Tsingke Biotechnology	/	Forward: 5'-TGATTGTCGAAGAACCATTTCGG-3' Reverse: 5'-TGCAGGGATCAAAAAGTTTGGAG-3

Supplementary Table 4. The adjacent nodes of DEFERGs.

#node	Identifier	Node_degree
PTEN	9606.ENSP00000361021	17
IFNG	9606.ENSP00000229135	10
SQSTM1	9606.ENSP00000374455	9
CAV1	9606.ENSP00000339191	8
MAPK1	9606.ENSP00000215832	8
PIK3CA	9606.ENSP00000263967	8
ADIPOQ	9606.ENSP00000389814	6
MDM4	9606.ENSP00000356150	6
NOX4	9606.ENSP00000263317	6
PRKAA1	9606.ENSP00000346148	6
ULK1	9606.ENSP00000324560	6
IDO1	9606.ENSP00000430950	5
TSC1	9606.ENSP00000298552	5
USP7	9606.ENSP00000343535	5
DLD	9606.ENSP00000205402	4
GABARAPL1	9606.ENSP00000411256	4
IFNA2	9606.ENSP00000369554	4
LAMP2	9606.ENSP00000408411	4
NOS2	9606.ENSP00000327251	4
NOX1	9606.ENSP00000362057	4
HMGB1	9606.ENSP00000345347	3
PLIN2	9606.ENSP00000276914	3
TERT	9606.ENSP00000309572	3
ZEB1	9606.ENSP00000354487	3
CA9	9606.ENSP00000367608	2
CDC25A	9606.ENSP00000303706	2
CS	9606.ENSP00000342056	2
FGF21	9606.ENSP00000471477	2
IFNA21	9606.ENSP00000369574	2
NOX5	9606.ENSP00000373518	2
ALOX12	9606.ENSP00000251535	1
ALOX15B	9606.ENSP00000369530	1
AQP5	9606.ENSP00000293599	1
AQP8	9606.ENSP00000219660	1
ASAH2	9606.ENSP00000378897	1

BRD2	9606.ENSP00000378702	1
BRPF1	9606.ENSP00000373340	1
DPEP1	9606.ENSP00000376807	1
FZD7	9606.ENSP00000286201	1
GLS2	9606.ENSP00000310447	1
MIB2	9606.ENSP00000426103	1
MTDH	9606.ENSP00000338235	1
RBMS1	9606.ENSP00000294904	1
SIAH2	9606.ENSP00000322457	1
SMPD1	9606.ENSP00000340409	1
SUV39H1	9606.ENSP00000337976	1
TIMM9	9606.ENSP00000378588	1
ALOXE3	9606.ENSP00000314879	0
AMN	9606.ENSP00000299155	0
CHAC1	9606.ENSP00000484644	0
ETV4	9606.ENSP00000321835	0
HCAR1	9606.ENSP00000389255	0
MIOX	9606.ENSP00000216075	0
MS4A15	9606.ENSP00000386022	0
OSBPL9	9606.ENSP00000412733	0
PANX2	9606.ENSP00000379183	0
POM121L12	9606.ENSP00000386133	0
TFR2	9606.ENSP00000420525	0
YTHDC2	9606.ENSP00000161863	0
YY1AP1	9606.ENSP00000357323	0
