# Supplementary material for: Identification and validation of the biomarkers related to ferroptosis in calcium oxalate nephrolithiasis
Source: Aging (Albany NY). 2024 Mar 25;16(7):5987–6007. doi: 10.18632/aging.205684 (PMC11042938; doi:10.18632/aging.205684)
Supplement: Supplementary Table 6 [file aging-16-205684-s005.pdf]

Supplementary Table 6. Two clusters in Plaque group based on the hub DEFERGs.

ID	Cluster
GSM1900676_Plaque	C1
GSM1900678_Plaque	C1
GSM1900680_Plaque	C1
GSM1900681_Plaque	C1
GSM1900683_Plaque	C2
GSM1900687_Plaque	C1
GSM1900689_Plaque	C2
GSM1900691_Plaque	C2
GSM1900693_Plaque	C2
GSM1900695_Plaque	C2
GSM1900697_Plaque	C2
GSM1900699_Plaque	C2
GSM1900701_Plaque	C2
GSM1900704_Plaque	C2
GSM1900706_Plaque	C1
GSM1900708_Plaque	C1
GSM1900709_Plaque	C2
GSM1900711_Plaque	C2
GSM1900712_Plaque	C2
GSM1900715_Plaque	C2
GSM1900717_Plaque	C2
GSM1900719_Plaque	C1
GSM1900721_Plaque	C1
GSM1900723_Plaque	C2
GSM1900725_Plaque	C2
GSM1900727_Plaque	C1
GSM1900729_Plaque	C2
GSM1900731_Plaque	C2
GSM1900734_Plaque	C2
